# Impact on clinical practice of updated guidelines on iodinated contrast material: CINART

**DOI:** 10.1007/s00330-020-06719-7

**Published:** 2020-02-27

**Authors:** E. C. Nijssen, P. J. Nelemans, R. J. Rennenberg, A. J. van der Molen, G. V. van Ommen, J. E. Wildberger

**Affiliations:** 1 Department of Radiology & Nuclear Medicine, Maastricht University Medical Centre, PO Box 5800, 6202 AZ Maastricht, The Netherlands; 2 Department of Epidemiology, Maastricht University, PO Box 616, 6200 MD Maastricht, The Netherlands; 3 Department of Internal Medicine, Maastricht University Medical Centre, PO Box 5800, 6202 AZ Maastricht, The Netherlands; 4 Department of Radiology, Leiden University Medical Centre, Postal zone C-2S, 2333 ZA Leiden, The Netherlands; 5 Department of Cardiology, Maastricht University Medical Centre, PO Box 5800, 6202 AZ Maastricht, The Netherlands

**Keywords:** Clinical practice guideline, Contrast media, Preventive measures, Acute kidney injury, Costs and cost analysis

## Abstract

**Objective:**

Guidelines on safe use of iodinated contrast material recommend intravenous prophylactic hydration to prevent post-contrast adverse (renal) effects. Recently, guidelines have been updated and standard prophylaxis is no longer recommended for the majority of patients. The current study aims to evaluate the consequences for clinical practice of the updated guidelines in terms of complications, hospitalisations, and costs.

**Methods:**

The Contrast-Induced Nephropathy After Reduction of the prophylaxis Threshold (CINART) project is a retrospective observational study. All elective procedures with intravascular iodinated contrast administration at Maastricht University Medical Centre (UMC+) in patients aged > 18 years, formerly eligible for prophylaxis (eGFR 30–44 ml/min/1.73 m^2^ or eGFR 45–59 ml/min/1.73 m^2^ in combination with diabetes or > 1 predefined risk factor), and currently eligible for prophylaxis (eGFR < 30 ml/min/1.73 m^2^) were included. Data were used to calculate relative reductions in complications, hospitalisations, and costs associated with standard prophylactic intravenous hydration. CINART is registered with Clinicaltrials.gov: NCT03227835.

**Results:**

Between July 1, 2017, and July 1, 2018, 1992 elective procedures with intravascular iodinated contrast in patients formerly and currently eligible for prophylaxis were identified: 1808 in patients formerly eligible for prophylaxis and 184 in patients currently eligible for prophylaxis. At Maastricht UMC+, guideline updates led to large relative reductions in numbers of complications of prophylaxis (e.g. symptomatic heart failure; − 89%), extra hospitalisations (− 93%), and costs (− 91%).

**Conclusion:**

Guideline updates have had a demonstrable impact on daily clinical practice benefiting patient, hospital, and health care budgets. Clinical practice varies between institutions and countries; therefore, a local estimation model is provided with which local impact on costs, hospitalisations, and complications can be calculated.

**Key Points:**

*• Clinical practice guidelines recommend prophylactic intravenous hydration to prevent post-contrast adverse outcomes such as contrast-induced acute kidney injury.*

*• Clinical practice guidelines have recently been updated, and standard prophylaxis is no longer recommended for the majority of patients.*

*• The guideline updates have a large impact on daily clinical practice: relative reductions at Maastricht UMC+ were − 89% prophylaxis complications, − 93% hospitalisations, and − 91% costs, and similar reductions are expected for Dutch and adherent European medical centres.*

**Electronic supplementary material:**

The online version of this article (10.1007/s00330-020-06719-7) contains supplementary material, which is available to authorized users.

## Introduction

Guidelines on safe use of iodinated contrast material recommend intravenous prophylactic hydration to prevent post-contrast adverse (renal) effects [1–5]. The AMACING (A MAastricht Contrast-Induced Nephropathy Guideline) trial showed that standard prophylaxis was not effective in the majority of patients targeted by the guidelines, a result which was confirmed by 1-year follow-up data [6, 7]. Benefits of standard prophylactic intravenous hydration, such as a reduction in post-contrast acute kidney injury and/or long-term adverse effects, were not found. The main differences between randomised groups with and without prophylaxis were rates of complications after prophylaxis (5.5% vs 0.0%) and costs (€1455 vs €792). The latter difference was mainly due to the hospitalisation required for prophylaxis, which is also the main burden for patients and hospitals associated with prophylaxis.

Recently guidelines have been updated, and standard prophylaxis is no longer routinely recommended for patients like those who participated in the AMACING trial (i.e. with estimated glomerular filtration rate (eGFR) 30–59 ml/min/1.73 m^2^ combined with risk factors) [2, 3, 5, 8–10]. The changes in the recommendations on standard prophylaxis for elective patients in the Dutch (The Radiological Society of The Netherlands, NVvR) and European (European Society of Urogenital Radiology, ESUR) guidelines are summarised in Table 1.

**Table 1:** Clinical practice recommendations for elective patients before and after guideline updates

European guideline recommendation*	Before January 2018 update	After January 2018 update
Patient eligible for standard prophylaxis	eGFR < 45 ml/min/1.73 m^2^ with iv contrast eGFR < 60 ml/min/1.73 m^2^ with ia contrast	eGFR < 30 ml/min/1.73 m^2^ (eGFR < 45 ml/min/1.73 m^2^ for intra-arterial contrast with first pass renal exposure)
Standard prophylaxis	iv 0.9% NaCl at least 6 h before and after or iv 1.4% NaHCO_3_ 1 h before and 6 h after	iv 0.9% NaCl 3 to 4 h before and 4 to 6 h after or iv 1.4% NaHCO_3_ 1 h before (and 4 to 6 h after for intra-arterial contrast with first pass renal exposure)
Dutch guideline recommendation^§^	Before November 2017 update	After November 2017 update
Patient eligible for standard prophylaxis	eGFR < 45 ml/min/1.73 m^2^ or eGFR 45–59 ml/min/1.73 m^2^ combined with diabetes or > 1 risk factor^$^	eGFR < 30 ml/min/1.73 m^2^
Standard prophylaxis	iv 0.9% NaCl 4 or 12 h before and 4 or 12 h after^#^	iv 1.4% NaHCO_3_ 1 h before (optional: 6 h after)

*iv*, intravenous; *ia*, intra-arterial; *eGFR*, estimated glomerular filtration rate. *ESUR guidelines on contrast media, versions 9 and 10. ^§^Centraal Begeleidings Orgaan guideline on iodinated contrast material 2007 [11], and The Radiological Society of The Netherlands (RSTN - NVvR) [2] guideline on safe use of contrast media 2017. ^$^Age > 75 years, anaemia, cardiovascular disease, nephrotoxic medication. ^#^The guidelines recommended two standard hydration protocols: a short protocol (4 h pre- and 4 h post-hydration) and a long protocol for patients with cardiac or renal failure (12 h pre- and 12 h post-hydration with reduced flow rate)

After the in-house protocol had been updated in accordance with the recent guideline updates, the observational Contrast-Induced Nephropathy After Reduction of the prophylaxis Threshold (CINART) project was started with the aim to evaluate consequences for clinical practice at Maastricht University Medical Centre (Maastricht UMC+). The AMACING trial showed that abolishing prophylaxis in this patient population (i.e. eGFR 30–44 ml/min/1.73m^2^ or eGFR 45-59 ml/min/1.73 m^2^ combined with diabetes or >1 risk factors) did not lead to changes in renal adverse events: the only changes would be in incidences of complications, hospitalisations, and costs. We therefore evaluated the impact of the guideline updates on clinical practice in those terms: patient burden (complications of prophylaxis), hospital burden (extra hospitalisations for prophylaxis), and costs [12].

Because the clinical practice of giving prophylaxis varies across countries and between hospitals, a local estimation model was construed by which local impact on complications, hospitalisations, and costs may be calculated.

## Materials and methods

### Study design, participants, and data collection

CINART is a 1-year retrospective observational study (Clinicaltrials.gov NCT03227835) carried out after prophylaxis was abolished for patients such as those who participated in the AMACING trial.

All elective procedures with intravascular iodinated contrast administration at Maastricht UMC+ in patients aged 18 years or over and formerly eligible for prophylaxis (similar to the inclusion criteria of the AMACING trial, i.e. with eGFR 30–44 ml/min/1.73 m^2^; or with eGFR 45–59 ml/min/1.73 m^2^ in combination with diabetes or > 1 of the following predefined risk factors: age > 75 years, anaemia, cardiovascular disease, nephrotoxic medication) [1, 11] or currently eligible for prophylaxis (i.e. with eGFR < 30 ml/min/1.73 m^2^) [2, 8] were eligible for inclusion.

The data concern procedures; therefore, repeat inclusion of patients was allowed. Data were retrospectively collected from patient electronic files. The Medical Research Ethics Committee Maastricht UMC+ waived the requirement for informed consent.

CINART is the core study for this manuscript, but the calculations are based on CINART and two other previously published studies [6, 7, 13].

1. The AMACING trial [6, 7] was a randomised controlled non-inferiority trial in elective patients with eGFR 30–44 ml/min/1.73 m^2^ or eGFR 45–59 ml/min/1.73 m^2^ combined with risk factors. The trial compared patients receiving standard prophylactic intravenous hydration with normal saline to patients not receiving prophylaxis. The differences between groups were in complications after prophylaxis (5.5% vs 0.0%) and costs (€1455 vs €792).
2. The observational study in eGFR < 30 ml/min/1.73 m^2^ patients [13]. This study contains 4 years’ worth of data of all elective procedures with intravascular contrast carried out at our centre. Results showed 6.4% complications in patients receiving prophylaxis; no similar events were registered in the no prophylaxis group. Data on complications of prophylaxis was obtained from the medical records, and similar entries were not observed around the time of the contrast procedure in patients without prophylaxis.

Hydration protocols are given in Table 1.

### Outcomes

Primary outcome of CINART was the number of elective radiology or cardiology procedures in patients (no longer) eligible for standard prophylaxis, i.e. the number of procedures in patients eligible for standard prophylaxis according to guidelines before the update, and the number of procedures in patients eligible for standard prophylaxis according to guidelines after the update. Additional information concerns the proportions of outpatients, defined as the proportion of patients not hospitalised at the moment of referral for the contrast procedure. The results were subsequently used to calculate the main results of the current study: the impact of guideline updates in terms of relative reduction in the numbers of complications, hospitalisations, and costs associated with prophylactic intravenous hydration.

The reported change in the rate of complications is based on the observed numbers of procedures in the CINART study and the observed rates of complications in the AMACING trial.

The change in the number of hospitalisations is based on observed numbers from the CINART study.

The change in costs is based on the observed numbers of procedures in the CINART study and the prospective data on cost difference from the AMACING trial.

### Calculations

Calculations were based on numbers registered in the CINART project and estimates of complication rates and costs from previous studies. The prospective AMACING trial and our 4-year observational study on elective patients with eGFR < 30 ml/min/1.73 m^2^ provided estimates of complication rates in formerly eligible and currently eligible patients, respectively [6, 13]. Data on mean costs of resource use in patients receiving standard prophylaxis are from the AMACING study [6].

The change in the number of complications was calculated as follows:

**Table:** 

*Complications avoided* after guideline update	=	Incidence of complications in patients formerly eligible for prophylaxis	x	Number of procedures in patients formerly eligible for prophylaxis	x	Adherence to guideline recommendations

The change in the number of hospitalisations for prophylaxis was calculated as follows:

**Table:** 

*Beds freed* after guideline update	=	Number of procedures in patients formerly eligible for prophylaxis	x	% outpatients in patients formerly eligible for prophylaxis	x	Adherence to guideline recommendations

The change in costs associated with elective contrast procedures after the guideline update was calculated as follows:

**Table:** 

*Cost savings* after guideline update	=	Number of procedures in patients formerly eligible for prophylaxis	x	Extra costs of resources used by patients receiving standard prophylaxis	x	Adherence to guideline recommendations

### Role of the funding source

The funder, Stichting de Weijerhorst, was not involved in study design, patient recruitment, data collection, analysis, interpretation or presentation, writing or editing of the reports, or the decision to submit for publication. The corresponding author had full access to all data in the study and had final responsibility for the decision to submit for publication.

## Results

The in-house protocol for safe use of iodinated contrast material was updated and implemented in the summer of 2017 at Maastricht UMC+, after which only patients with eGFR < 30 ml/min/1.73 m^2^ were eligible for standard prophylactic intravenous hydration.

**Fig. 1:**
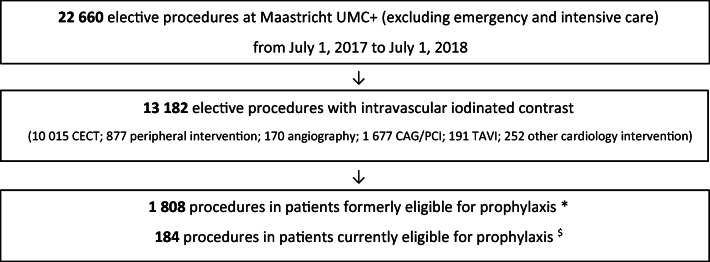
Screening and inclusion profile. CECT, contrast-enhanced computed tomography; CAG, coronary angiography; PCI, percutaneous coronary intervention; TAVI, transcatheter aortic valve implantation. *i.e. patients with eGFR 30–59 ml/min/1.73 m^2^ combined with risk factors; $ i.e. patients with eGFR < 30 ml/min/1.73 m^2^

From July 1, 2017, until July 1, 2018, a total of 1992 elective procedures with intravascular iodinated contrast material in patients formerly and currently eligible for prophylaxis were identified: 1808 procedures in patients formerly eligible for prophylaxis (with eGFR 30–59 ml/min/1.73 m^2^ combined with risk factors) and 184 procedures in patients with eGFR < 30 ml/min/1.73 m^2^ currently eligible for prophylaxis (Fig. 1).

**Fig. 2:**
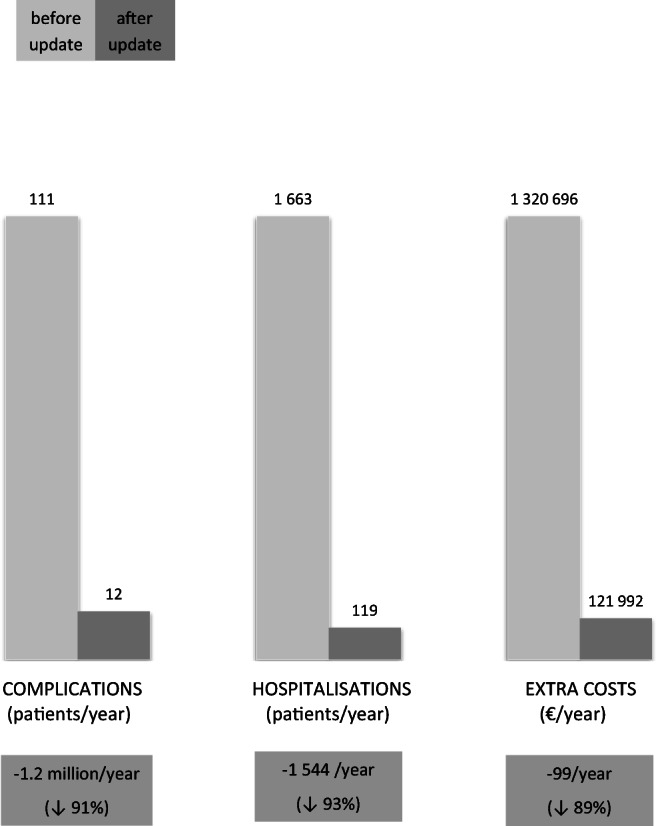
Complications, hospitalisations, and costs associated with standard prophylaxis at Maastricht UMC+ before and after guideline updates

Complications, hospitalisations, and costs associated with standard prophylaxis before and after the guideline updates are illustrated in Fig. 2; an interactive calculating tool is presented in Table 2.

**Table 2:** Local estimation model: complications, hospitalisations, and extra costs associated with standard prophylaxis before and after guideline updates

	Input parameter		CINART	Interactive calculator
				INPUT
	GENERAL			Enter local parameters*
I1	Elective procedures/year at location		22.660	75.000.000
I2	Proportion elective procedures with intravascular contrast administration (%)		58.17%	100.00%
I3	Extra costs per prophylaxis patient ^$^		€ 663	€ 663,00
I4	Adherence to guidelines (%) ^Ɨ^		100%	40.00%
	IN PATIENTS FORMERLY ELIGIBLE FOR PROPHYLAXIS**			
I5	Elective procedures (%)		13.72%	13.72%
I6	Proportion of outpatients (%)		85.40%	85.40%
I7	Rate of complications after prophylaxis		–	5.50%
	IN PATIENTS CURRENTLY ELIGIBLE FOR PROPHYLAXIS***			
I8	Elective procedures (%)		1.40%	1.40%
I9	Proportion of outpatients (%)		64.67%	64.67%
I10	Rate of complications after prophylaxis		–	6.40%
	Resultant parameter	Formula		Interactive calculator OUTPUT
R1	Elective procedures with iv or ia contrast administration/year	I1 x I2	13.182	75.000.000
R2	Procedures in patients formerly eligible for prophylaxis /year	R1 x I5	1.808	10.287.000
R3	Procedures in patients currently eligible for prophylaxis/year	R1 x I8	184	1.046.250
	COMPLICATIONS			
R4	Complications/year before guideline update	(R2 x I7) + (R3 x I10) x I4	111	253.098
R5	Complications/year after guideline update	(R3 x I10) x I4	12	26.784
R6	Complications/year avoided after guideline update	(R2 x I7) x I4	99	226.314
	HOSPITALISATIONS			
R7	Extra hospitalisations for prophylaxis/year before guideline update	[(R2 x I6) + (R3 x I9)] x I4	1.663	3.784.683
R8	Extra hospitalisations for prophylaxis/year after guideline update	(R3 x I9) x I4	119	270.644
R9	Beds freed/year after guideline update	(R2 x I6) x I4	1.544	3.514.039
	COSTS			
R10	Total extra costs/year before guideline update	[(R2 + R3) x I3] x I4	€ 1.320.696,00	€ 3.005.577.900,00
R11	Total extra costs/year after guideline update	(R3 x I3) x I4	€ 121.992,00	€ 277.465.500,00
R12	Cost savings/year after guideline update	(R2 x I3) x I4	€ 1.198.704,00	€ 2.728.112.400,00

*iv*, intravenous; *ia*, intra-arterial; *I*, input; *R*, result

*Local values can be inserted in the online version of the calculator (see supplementary material). Here an example calculation for the estimated 75 million injections with iodinated contrast each year worldwide (in 2005) is shown, with adherence to guideline recommendations set at 40%. **Formerly eligible = with eGFR < 45 ml/min/1.73 m^2^ or with eGFR 45–59 ml/min/1.73 m^2^ combined with diabetes/ > 1 risk factor; ***Currently eligible = with eGFR < 30 ml/min/1.73 m^2^

^$^Default is set at the mean difference in costs in euros up to 1 month post-contrast between prophylaxis and no prophylaxis patients as registered in AMACING. The AMACING trial showed that costs are primarily incurred by (extra) hospitalisation for prophylaxis: local hospitalisation costs can be inserted

^Ɨ^Adherence is set at 100% for the Netherlands, since the authorities imposed guideline recommendations quite strictly. Regulations may differ in different countries: local adherence can be inserted

In the calculations for Maastricht UMC+ below, adherence to guideline recommendations is set at 100%.

### Complications of prophylaxis

The number of complications of prophylaxis was calculated based on the 5.5% rate of complications found in AMACING trial patients with eGFR 30–59 ml/min/1.73 m^2^ combined with risk factors, and the 6.4% rate of complications found in our 4-year observational study in patients with eGFR < 30 ml/min/1.73 m^2^ [6, 13].

Total complications before update: (1808 x 0.055) + (184 x 0.064) = 111/year

Total complications after update: 184 x 0.064 = 12/year

*Total complications avoided* after guideline update: 0.055 x 1808 = 99/year (− 89%)

### Hospitalisation for prophylaxis

CINART registered 85.4% outpatients (1544/1808) in the group formerly eligible for prophylaxis, and 64.7% outpatients in the group currently eligible for prophylaxis (119/184).

Total extra hospitalisations before update: (1808 x 0.854) + (184 x 0.647) = 1663/year

Total extra hospitalisations after update: 184 x 0.647 = 119/year

*Total beds freed* after the guideline update: 1808 x 0.854 = 1544/year (− 93%)

### Costs

Cost calculations were based on the difference in costs associated with elective contrast procedures (excluding costs of the procedure itself) up to 1 month post-contrast as registered in the AMACING trial [6]: mean extra costs of resources used by patients receiving standard prophylaxis were €663 per procedure per patient. These costs were mostly due to hospitalisation costs.

Total extra costs before the guideline update: 1992 x €663 = €1,320,696/year

Total extra costs after the guideline update: 184 x €663 = €121,992/year

*Total savings* after the guideline update: €1,198,704/year (− 91%)

### First pass renal exposure

As shown in Table 1, the European guidelines recommend a higher threshold for standard prophylaxis if there is first pass renal exposure (i.e. if contrast reaches the renal arteries more or less undiluted as is the case after intra-arterial catheter injections in the left heart, thoracic aorta, suprarenal abdominal aorta, and after direct injection into renal arteries). For such procedures, the European guidelines recommend prophylaxis for all patients with eGFR < 45 ml/min/1.73 m^2^.

In patients with an eGFR between 30 and 45 ml/min/1.73m^2^, CINART identified only 79 procedures where first pass renal exposure was a possibility (procedures such as peripheral angiography/intervention, transcatheter aortic valve implantation (TAVI) and endovascular aneurysm repair (EVAR)). Thus 0.3% (79/22660) of all elective procedures, and 0.6% (79/13182) of elective procedures with intravascular iodinated contrast administration, may possibly have been eligible for the higher threshold for prophylaxis recommended in the European guidelines.

### Local estimation model

The current study concerns data from Maastricht UMC+ and assumes 100% adherence to guideline recommendations. Local parameters may vary; therefore, local changes in costs, hospitalisations, and complications associated with prophylaxis can be estimated using the calculator in Table 2: the input section allows for local parameters to be inserted (see supplementary material for online version of the calculator).

Default values are set to the results of the current study: 58.2% elective radiology and cardiology procedures were with intravascular iodinated contrast administration; 13.7% of elective procedures with intravascular contrast were done in patients formerly eligible for prophylaxis, and 1.4% in patients currently eligible for prophylaxis; percentage outpatients was 85.4% in formerly eligible patients and 64.7% in currently eligible patients.

The AMACING trial showed that costs are primarily incurred by the required (extra) hospitalisation for prophylaxis: for outpatients without prophylaxis, extra costs up to 1 month post-contrast were near to zero [6]. Thus, in an outpatient setting, costs are expected to decrease in direct proportion to the number of patients no longer eligible for prophylaxis. Local costs of 8–24-h hospitalisation can be inserted in Table 2 to give a more exact estimation of local costs and savings (see supplementary material for online version of the calculator).

## Discussion

Abolishing standard prophylaxis for elective patients with eGFR 30–59 ml/min/1.73 m^2^ combined with risk factors and administering it only to elective patients with eGFR < 30 ml/min/1.73 m^2^ has led to estimated relative reductions of 89% in the number of patients suffering complications of prophylaxis such as symptomatic heart failure (99 cases a year); 93% in the number of hospitalisations for prophylaxis (1544 a year); and 91% in costs (€ 1.2 million a year) at Maastricht UMC+.

The current article is not about efficacy, appropriateness, risk, or benefit of intravenous prophylactic hydration. Neither is it about the risk of post-contrast AKI. Instead, the aim is to give insight into the impact on clinical practice of the recent updated guidelines on iodinated contrast material. Based on the current data, it is expected that patient and hospital burden are much reduced at institutions adhering to the guideline recommendations.

The current article focuses on the elective population for whom guidelines previously recommended standard prophylaxis and for whom standard prophylaxis has now been abolished. It is these patients for whom the guideline updates represent the greatest change (guideline recommendations deviate for acute situations), and it is these patients that represent the bulk of patients receiving standard prophylaxis before the guideline updates. Therefore, it is in this population that the impact for hospitals and health care budgets is found. Furthermore, assuming adherence to guideline recommendations, this impact on clinical practice exists irrespective of whether this population is truly at risk of post-contrast renal adverse events or not.

There are some limitations. The current study focuses on the Dutch guideline and the umbrella European (ESUR) guideline because they are paramount to our centre, but other guidelines and guideline-changes exist [3–6]. Currently, the two other prominent umbrella guidelines (North America – ACR, and Oceania – RANZCR) have been updated to uniformly recommend prophylaxis for patients with eGFR < 30 ml/min/1.73 m^2^ only [3, 5]. Other guidelines, such as the Asian and Canadian guidelines, have not been updated, but it is expected that individual countries will follow the European and/or American guidelines in determining local protocols.

Contrary to the updated Dutch guideline, the updated ESUR guidelines contain an extra recommendation for contrast procedures with first pass renal exposure: for such procedures European guidelines recommend prophylaxis for all patients with eGFR < 45 ml/min/1.73 m^2^ [8]. CINART identified 79 (0.3%) elective radiology and cardiology procedures in patients with an eGFR between 30 and 45 ml/min/1.73 m^2^ where first pass renal exposure is a possibility, although actual first pass renal exposure is only expected to have occurred in a small minority of these. Even if a greater portion involved first pass renal exposure, however, the effect on estimated impact is probably nil. This is because patients are normally hospitalised for this type of procedure, and thus, the number of extra hospitalisations for prophylaxis will not be much affected. The AMACING trial showed that costs of resources used by patients receiving standard prophylaxis are mainly hospitalisation costs [6], and therefore, costs will remain more or less unchanged. As for complications of prophylaxis, a worst-case scenario would be 4 extra complications a year (79*5.5%), from 0.09 to 0.12% per 13,182 elective procedures with intravascular iodinated contrast administration. Centres specialising in such procedures may wish to include patients with eGFR < 45 ml/min/1.73 m^2^ in the ‘currently eligible for prophylaxis’ group for their calculations in Table 2.

At this time, whereas American and Oceania guidelines still recommend standard intravenous hydration with normal saline 12 h before and 12 h after contrast administration [3, 5], the Dutch and European guidelines recommend or include an alternative in the form of intravenous sodium bicarbonate 1 h pre- and an optional 6 h post-contrast [2, 9]. The current study has not included the latter protocol in the calculations. However, in the absence of mitigating strategies, incidences of complications would likely be minimally affected—although there is not much data available in the literature, existing data appears to indicate that complication rates are similar for both types of prophylaxis [2]. Furthermore, we do not expect the shorter hydration protocol to further reduce hospitalisations and costs, unless centres opt for 1-h outpatient pre-hydration. There is little evidence of efficacy of different prophylaxis protocols, but it is thought that any effect of intravenous hydration in the prevention of renal injury is likely to be rate-dependent, which requires that the infusion be maintained throughout the period of contrast excretion by the kidney [14]. Taken together with the fact that the population currently eligible for prophylaxis is truly at risk [15], most centres will probably opt for a longer inpatient hydration protocol. Regardless, the effect on costs will be limited, because the population currently eligible for prophylaxis represents a fraction of the eligible population before the guideline update [15], and further reduction in hospitalisations within this population will therefore also represent only a fraction of the total.

The data presented was collected at a single centre. Because Maastricht UMC+ is both a secondary and tertiary referral centre and a community hospital, we expect its elective population and procedures to be representative of other Dutch and Western European academic medical centres. The results of the current study are especially useful because, where trials perforce report in numbers of unique patients, this study gives an indication of the various proportions of procedures with iodinated contrast administration to which guideline recommendations and extra hospitalisations apply. Furthermore, using the local estimation model will make the current information generally applicable to determining the impact of guideline updates on local clinical practice.

Parameters such as adherence to guideline recommendations, complication rates, and previous prophylaxis thresholds may vary amongst medical centres, which is why these were incorporated as variable input factors into the local estimation model. Guideline recommendations were imposed quite strictly in the Netherlands. This is why adherence is close to 100%, whereas experience and surveys have shown that elsewhere adherence may be absent (e.g. a hospital in China, personal communication) or somewhere at the level of 64–87% (e.g. ESUR guideline survey, personal communication) [16].

The rate of complications found in the AMACING trial is expected to be representative of the rate that occurs when adhering to the guideline recommendation for standard prophylaxis. At Maastricht UMC+, best clinical practice is followed, separate protocols with low flow rates were in place for patients with cardiac or renal failure (see Table 1), and patients at high risk of complications are not given prophylactic intravenous hydration. Furthermore, other papers reporting complications of intravenous fluids yield similar rates [2]. However, we are aware of the emergence of mitigating strategies that may help to avoid complications of intravenous hydration [17]. Because complication rates may vary between countries and centres, and successful strategies to mitigate the incidence of heart failure due to prophylaxis may be in place, the local estimation model also allows for insertion of differing incidences of complications.

Because local parameters will deviate, and for situations where detailed local parameters are known, a calculation model was included with which each medical centre may calculate local changes in costs, hospitalisations, and complications (Table 2). Some centres may have a larger proportion of inpatients for example, which is expected to affect the impact on costs and hospitalisations.

The presented local estimation model allows for the insertion of local parameters on elective procedures with intravascular contrast administration, extra costs per patient for prophylaxis, the level of adherence to guideline recommendations, proportion of procedures in patients formerly and currently eligible for prophylaxis, proportions patients formerly and currently eligible for prophylaxis that are outpatients, and incidences of complications in patients formerly and currently eligible for prophylaxis. Default parameters are set at the values found in the current study.

When inserting the number of iodinated contrast injections carried out worldwide in Table 2—estimated at 75 million a year in 2005 [18]—and assuming a worldwide average adherence to guideline recommendations of 40% (based on the reported 64–87% adherence in Europe, Oceania, and North America, and a worst-case scenario of zero adherence in Africa, South America, and half of Asia) [16, 18], the result estimates would be that over 225,000 patients a year no longer suffer from complications such as symptomatic heart failure associated with the prophylactic treatment, that over 3.5 million patients need no longer be hospitalised for prophylaxis, and that savings for health care budgets are over €2.7 billion, each year.

The recent updates of the guidelines on safe use of iodinated contrast material have a large impact on daily clinical practice, avoiding complications, freeing beds, and reducing costs. Local impact can be estimated using the local estimation model presented.

## Electronic supplementary material

ESM 1 (XLSX 13 kb)

## Abbreviations

AMACING: A MAastricht Contrast-Induced Nephropathy Guideline study
CINART: Contrast-Induced Nephropathy After the Reduction of the prophylaxis Threshold
eGFR: estimated Glomerular Filtration Rate
ESUR: European Society of Urogenital Radiology
NVvR: Nederlandse Verenging voor Radiologie (The Radiological Society of The Netherlands)
